# Making a voice heard: evaluation of a new service delivery in augmentative and alternative communication through qualitative interviews with people without natural speech

**DOI:** 10.1186/s13104-023-06310-5

**Published:** 2023-03-29

**Authors:** Anna Zinkevich, Sarah Anna Katharina Uthoff, Jens Boenisch, Stefanie Kalén Sachse, Helge Schnack, Carolin Garbe, Tobias Bernasconi, Lena Ansmann

**Affiliations:** 1 Department of Health Services Research, Carl von Ossietzky University of Oldenburg, Oldenburg, Germany; 2 Department of Special Education and Rehabilitation, University of Cologne, Cologne, Germany; 3 Institute of Medical Sociology, Health Services Research, and Rehabilitation Science (IMVR), Chair of Medical Sociology, University of Cologne, Cologne, Germany

**Keywords:** Qualitative interview study, Augmentative and alternative communication, Process evaluation, Health services research

## Abstract

**Objective:**

Due to communication barriers, people without natural speech who use augmentative and alternative communication (AAC) are rarely interviewed about their healthcare needs, expectations, and experiences. This qualitative interview study aims to investigate how AAC users evaluate a new service delivery (nSD) in AAC care in Germany.

**Results:**

We conducted 8 semi-structured qualitative interviews with 8 AAC users. The results of the performed qualitative content analysis show a positive evaluation of the nSD among AAC users. Contextual factors were identified that seem to hinder the achievement of the intervention goals. These include caregivers' prejudice and inexperience with AAC and an unfavourable environment in which AAC is used.

**Supplementary Information:**

The online version contains supplementary material available at 10.1186/s13104-023-06310-5.

## Introduction

Understanding individual preferences and needs of care recipients through qualitative interviewing is a widely used research method in health services research. But how can perceptions about care and individual experiences be obtained when interviewees have limited or no natural speech? Is it still possible to obtain valuable first-hand information for the evaluation of innovative care models?

Congenital and acquired disabilities are often associated with limitations in using natural speech. The group of people who rely on augmentative and alternative communication (AAC) is heterogeneous in terms of age, disability, and the extent of physical, intellectual, and communication impairments [1]. It is estimated that worldwide about 97 million people have either severe limitations in using natural speech or no intelligible natural speech at all [2]. AAC interventions include both non-electronic (e.g., symbol cards) and electronic aids (e.g., voice output devices). The provision of AAC is not clearly regulated in the German healthcare system. Utilisation and effectiveness of AAC is often limited due to problems in access to AAC, provision with inappropriate AAC aids, insufficient training of the therapeutic team, and unclear responsibilities of stakeholders involved [3–5]. To counteract these deficits, a new service delivery (nSD) was implemented and evaluated within the framework of the project “New Service-Delivery Model for Augmentative and Alternative Communication (AAC) Devices and Intervention”. In addition to standard care, the nSD includes case management, 4 AAC training sessions, and 20 AAC therapy sessions. The nSD was implemented in 3 participating AAC counselling centres in 3 different regions and was funded by the Innovation Fund of the Federal Joint Committee (G-BA) between December 2017 and August 2021. A more detailed description of the intervention and the study protocol can be found elsewhere [6]. Due to communicative barriers, AAC users are rarely interviewed about their healthcare needs and experiences [7]. This qualitative interview study aims to explore the questions of how AAC users evaluate the nSD, what contextual factors influence implementation and goal achievement, and what adaptation needs exist for the further development of the nSD.

## Main text

### Method

In November 2019, 8 semi-structured interviews were conducted with 8 different AAC users who participated in the nSD group. The participants were recruited in the three participating AAC counselling centres and the recruitment has followed the principle of purposeful sampling [8]. The selection criteria aimed to achieve a heterogeneous sample in terms of gender, age, and underlying condition/disability, as well as employment and living situation. Expert knowledge from AAC consultants from the participating counselling centres was consulted during preparation to adapt each interview to the interviewee's communication skills. The interview guideline was structured according to the research questions of the evaluation study, was pretested with a person using AAC and subsequently adapted (see Additional file 1) (Additional file 2) (Additional file 3). The age range of the interviewees was 34–58 years and 4 out of 8 interviewees were female. Interviewees had diverse physical, intellectual, and communication impairments. All interviewees had legal representatives who received study information in advance and signed the informed consent form. Prior to the interviews, the interviewees were also asked verbally for their consent. The interviews took place in sheltered workshops, residential homes and in a private appartment, lasted between 16:25 and 37:37 min and were conducted by two female researchers (AZ and SAKU) and a male student assistant (HS) who took the observation logs. Socio-demographic characteristics of the participants as well as information about the interview setting are provided as a table in (Additional file 1) (Additional file 2) (Additional file 3). Interviewees communicated using either the AAC aid received as part of the nSD, symbol cards provided by the research team, or with help of caregivers. Observation logs were taken and the interviews were audio-recorded.

### Data analysis

The recorded material was transcribed verbatim and pseudonymised by HS, AZ, and SAKU. A specific transcription guideline was developed which helped to merge data from the observation logs and transcripts. The transcripts were analysed by AZ and SAKU using the software MAXQDA Analytics Pro 2020 (version 20.3.0) and the method of qualitative content analysis according to Kuckartz [9]. The main categories were developed deductively based on the Medical Research Council (MRC) framework on process evaluations of complex interventions from Moore et al. [10]. The subcategories were formed inductively during the coding process of the first interview. AZ and SAKU discussed the developed category system in a consensus-building process before conducting the rest of the analysis independently with subsequent consensus building. In the process of forming inductive subcategories, on the one hand, new aspects were sought that were not represented by the deductively formed categories, and on the other hand, the aim was to achieve a greater differentiation of the deductive categories. According to the research question, the unit of analysis can be described as the entirety of 8 merged transcripts.

## Results

**Fig. 1:**
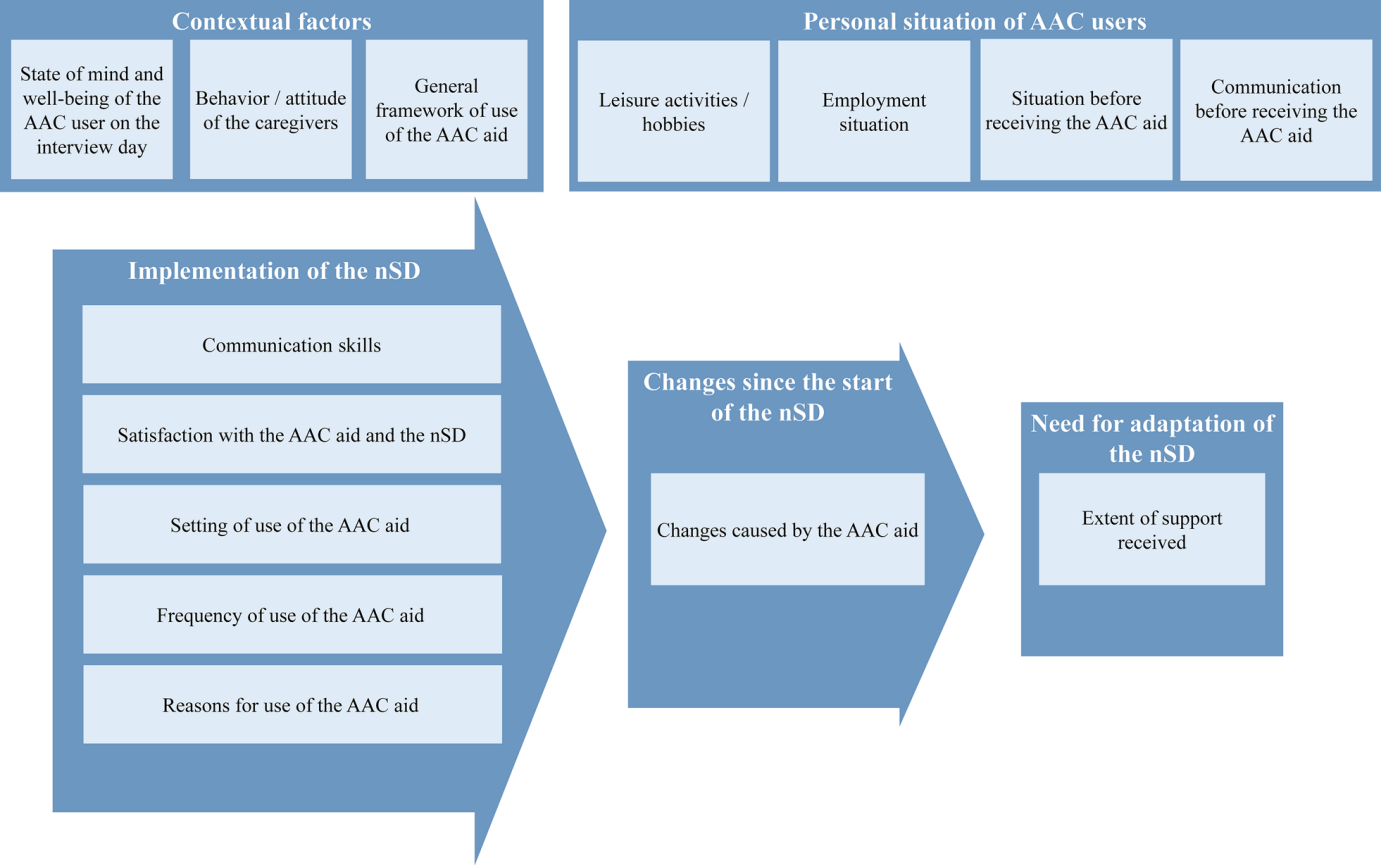
Category system

**Fig. 2:**
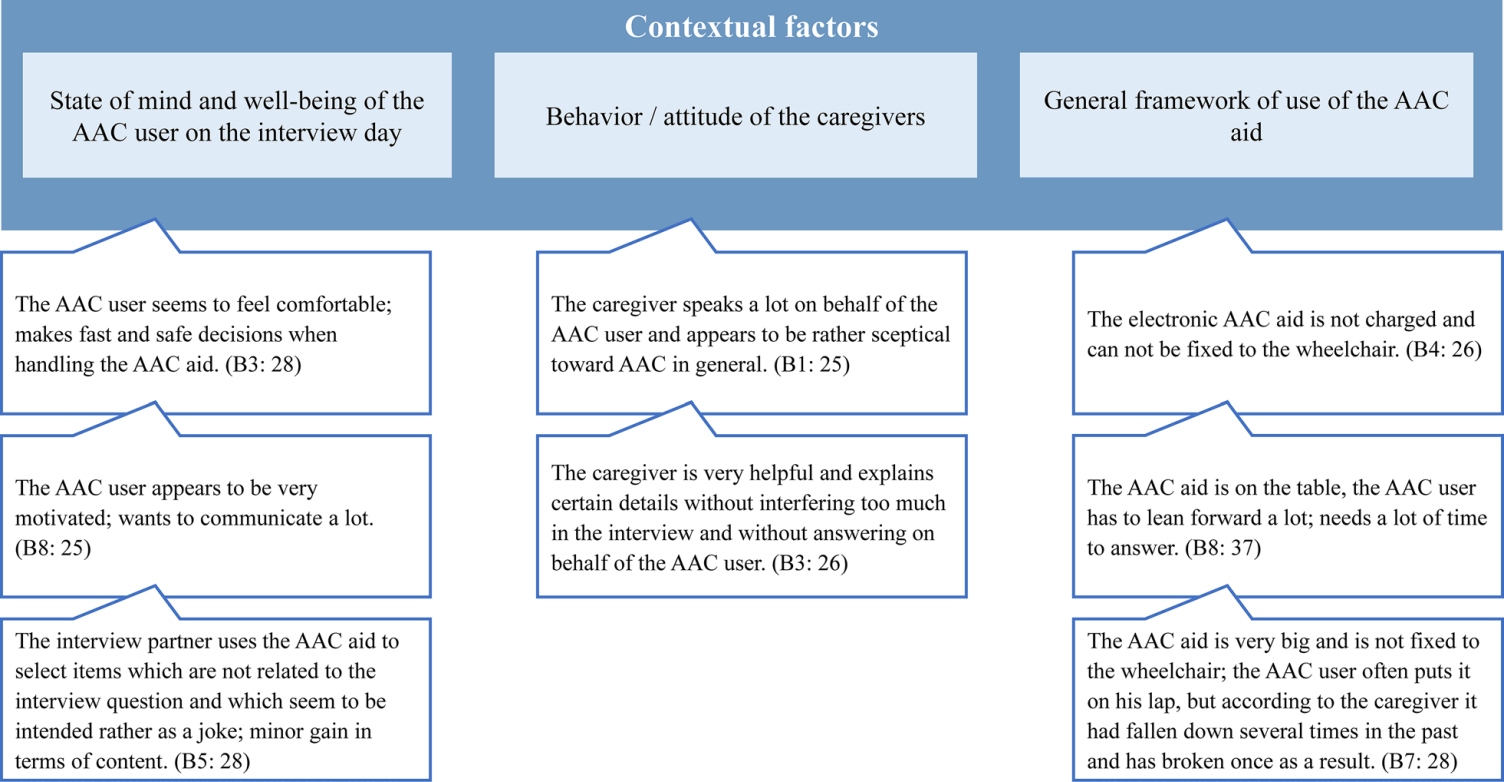
Category “Contextual factors” with sample codes. The sample codes in Fig. 2 are not verbatim quotes, but examples from the merged transcripts, which consist of the observation logs and the transcripts of the audio recordings

**Fig. 3:**
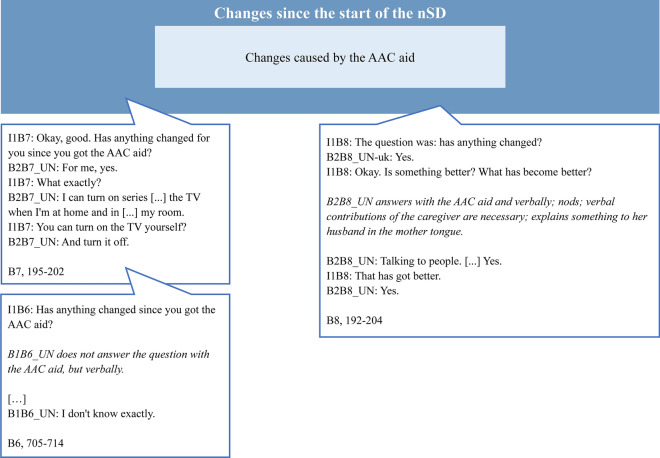
Category “Changes since the start of the nSD” with sample codes. I1B7: interviewer; B2B7_UN: AAC user, response with natural speech; I1B8: interviewer; B2B8_UN-uk: AAC user, response with the AAC aid; B2B8_UN: AAC user, response with natural speech

The main categories include personal background of AAC users, contextual factors, implementation of the nSD, changes since the beginning of the nSD, and the need for adaptation of the nSD. Figure 1 shows the complete category system. Participants tended to rate the nSD positively and reported improvements in communication skills, quality of life, and participation. The interviewees almost unanimously wished for more support (e.g., more therapy sessions). 6 out of 8 interviewees reported high satisfaction with the AAC aid, 2 were rather moderately satisfied. According to the interviewees, the AAC aid was predominantly used in the home setting and in the work context. Reasons for use often included leisure activities such as writing letters. Caregivers are described as an important contextual factor for regular and successful use, as they are often responsible for handling the AAC aid, e.g., charging. Through the nSD, participants reported to having developed new communication skills, such as placing orders in cafes, and having gained more independence in everyday life, such as operating the TV. Figures 2 and 3 demonstrate sample codes in the categories "Contextual factors" and "Changes since the start of the nSD". The second sample code in Fig. 3 is an illustration of how we combined verbal responses with observation logs.

## Discussion

The results of the interviews indicate a predominantly positive assessment of the nSD. Contextual factors could be identified that seem to hinder the achievement of the intervention's goals. These are, for example, caregivers' prejudices and inexperience regarding AAC, as well as unfavourable general conditions of the environment in which the AAC aid is used (Fig. 2). The results show that, from the perspective of the AAC users, nSD is indeed effective in improving communication skills. The results also demonstrate that improved communication skills are not the only benefit of the nSD; it also contributes to the facilitation of many daily activities that play a role in the AAC users' daily lives, which may lead to improved participation and a better quality of life. The underlying disabilities (e.g., with a progressive course) of the AAC users and the associated limitations that cannot be influenced by the nSD should always be considered as an important factor when discussing the results. However, the results of the study also show that the hindering contextual factors are not sufficiently addressed and that there is still a need for further development here. In their study investigating the communication between AAC users and healthcare professionals, Stans et al. [7] come to similar conclusions regarding the high importance of the communication context as well as the need for thorough preparation of dialogues with communication vulnerable people. Further development of the nSD could, for example, include training for the caregivers involved, addressing factors such as attaching the AAC aid to the wheelchair, charging electronic AAC aids and general information about AAC. Another adjustment should definitely be the increase in the number of therapy sessions. In line with our results, the study by Broomfield et al. [11] provides evidence that AAC users can not only contribute to answering evaluative research questions, as our results show, but can also be involved in earlier stages of the research process. Broomfield et al. [11] examined the involvement of people with communicative limitations in the context of participatory health services research and come to encouraging conclusions.

## Conclusions

The nSD was positively evaluated by AAC users. A variety of contextual factors could be identified which have an influence on the achievement of the nSD goals and which therefore need to be addressed in the further development of the nSD. Furthermore, AAC users can and should be included in health services research as they can provide valuable information on their healthcare needs and preferences.

### Limitations

Our study has certain limitations, which are briefly outlined below: (1) severe intellectual and motor limitations of some AAC users resulted in limited content gain in some interviews; (2) limited sample size. A larger qualitative study with a larger sample size would be essential for deeper insights; (3) we only interviewed AAC users from the intervention group and have no comparative qualitative data from AAC users experiencing standard AAC care in Germany; (4) we only interviewed adult AAC users, it would be important to also interview children and adolescents about their experiences with the nSD; (5) in order to obtain insightful research findings, it is important for the interviewers to prepare for various possible communication scenarios to be able to react adequately, flexibly and quickly to the communication strategies of the interview partner; (6) qualitative data obtained from other methods, such as ethnographic approaches or video analyses, would have extended the understanding of the impact of nSD.

## Supplementary Information

**Additional file 1: **Semi-structured interview guideline.

**Additional file 2: **Socio-demographic characteristics of the interview participants and information on the interview setting.

**Additional file 3: **Excerpt from the transcription guideline.

## Data Availability

The datasets are available from the corresponding author on reasonable request.

## Abbreviations

AAC: Augmentative and alternative communication
nSD: New service delivery (model)
