# Supplementary material for: Making a voice heard: evaluation of a new service delivery in augmentative and alternative communication through qualitative interviews with people without natural speech
Source: BMC Res Notes. 2023 Mar 29;16:42. doi: 10.1186/s13104-023-06310-5 (PMC10053108; doi:10.1186/s13104-023-06310-5)
Supplement: Supplementary file 1 — Additional file 1: Semi-structured interview guideline. [file 13104_2023_6310_MOESM1_ESM.docx]

**Supplementary material**

**Semi-structured interview guideline**

- What do you do often? (school, job, workshop, only at home, etc.)

- How were you doing before you got your AAC aid?

*If this question is not understood:*

How were you doing before (therapist name) came to see you?

OR

How are you doing without the AAC aid?

- How did you communicate before the project?

**- How satisfied are you with your new AAC aid since you started working with (therapist name)?**

- With whom do you use the AAC aid?

- Where do you use the AAC aid?

- How often do you use the AAC aid?

- Why do you use the new AAC aid?

**- Has anything changed as a result of the AAC aid?**

- What has become better?

- What has become worse?

**- Did you get enough help from (therapist name)?**

- Would you like more help?

- Have you had enough meetings?

- What else would you like to say?
