# Supplementary material for: Making a voice heard: evaluation of a new service delivery in augmentative and alternative communication through qualitative interviews with people without natural speech
Source: BMC Res Notes. 2023 Mar 29;16:42. doi: 10.1186/s13104-023-06310-5 (PMC10053108; doi:10.1186/s13104-023-06310-5)
Supplement: Supplementary file 2 — Additional file 2: Socio-demographic characteristics of the interview participants and information on the interview setting. [file 13104_2023_6310_MOESM2_ESM.docx]

**Supplementary material**

**Socio-demographic characteristics of the interview participants and information on the interview setting**

| **Interview participant** | **Age**  **(in years)** | **Sex** | **Disability / impairment** | **Type of AAC provided** | **Communication partner present in the interview** | **Interview location** | **Length of the interview**  **(in minutes)** |
| --- | --- | --- | --- | --- | --- | --- | --- |
| **B1** | 40–50 | Female | Down syndrome, Moyamoya disease | Aided AAC (electronic device) | Employee of the sheltered workshop | Sheltered workshop | 27 |
| **B2** | 30–40 | Male | General developmental disorder (not further specified) | Aided AAC (electronic device) | None | Residential home for people with disabilities | 32 |
| **B3** | 50–60 | Female | Cerebral palsy | Aided AAC (electronic device) | Caregiver in a residential home for people with disabilities | Residential home for people with disabilities | 19 |
| **B4** | 50–60 | Female | Cerebral palsy | Aided AAC (electronic device) | Caregiver in a residential home for people with disabilities | Residential home for people with disabilities | 33 |
| **B5** | 50–60 | Male | Cerebral palsy | Aided AAC (electronic device) | None | Residential home for people with disabilities | 23 |
| **B6** | 50–60 | Female | Down syndrome | Aided AAC (electronic device) | Caregiver in a residential home for people with disabilities | Residential home for people with disabilities | 19 |
| **B7** | 30–40 | Male | Traumatic brain injury | Aided AAC (electronic device) | Employee of the sheltered workshop | Sheltered workshop | 25 |
| **B8** | 50–60 | Male | Encephalitis | Aided AAC (electronic device) | Wife | Appartment | 37 |
