# Supplementary material for: Making a voice heard: evaluation of a new service delivery in augmentative and alternative communication through qualitative interviews with people without natural speech
Source: BMC Res Notes. 2023 Mar 29;16:42. doi: 10.1186/s13104-023-06310-5 (PMC10053108; doi:10.1186/s13104-023-06310-5)
Supplement: Supplementary file 3 — Additional file 3: Excerpt from the transcription guideline. [file 13104_2023_6310_MOESM3_ESM.docx]

**Supplementary material**

**Excerpt from the transcription guideline**

| always  absolutely | Emphasised word or syllable | Speech sound module |
| --- | --- | --- |
| Yes--- | Elongation |  |
| **never**  *never* | Loudly spoken word  Softly spoken word |  |
| usi- | Word break | Word breaks module |
| (clears throat) (sighs) (laughs)  "mhm" (affirmative) "mhm" (denying) | Non-verbal expressions | Non-speech events module |
| (knocks on the table) | Audible actions |  |
| (mobile phone ringing) | Background noises |  |
